# Religion, Acculturation, and Incarceration: Determinants of Substance Use among Hispanic Adults in the United States

**DOI:** 10.1155/2014/459596

**Published:** 2014-08-25

**Authors:** Benjamin J. Becerra, Monideepa B. Becerra, Miryam C. Gerdine, Jim E. Banta

**Affiliations:** ^1^School of Public Health, Loma Linda University, Loma Linda, CA 92350, USA; ^2^Department of Health Science and Human Ecology, California State University, San Bernardino, CA 92407, USA; ^3^Latino Caucus for Public Health, USA

## Abstract

*Objective*. The influence of religion, acculturation, and incarceration on substance abuse has been studied, though predominantly among adolescents. Little research exists on how such factors influence substance use among Hispanic adults. The objective of this study was to assess key determinants of substance use among Hispanic adults. *Methods*. Public access 2012 National Survey on Drug Use and Health was utilized. Univariate and multivariable logistic regression analyses were conducted while accounting for complex survey design to obtain population-weighted estimates. Receiver operator curve analysis was used to evaluate the relative contribution of each variable. *Results*. Importance of religious influence in life and Spanish language interview were associated with lower odds of substance use, while history of incarceration increased the likelihood of substance use among Hispanic adults. Other factors associated with lower odds were increasing age, being female, and currently married. Other factors associated with increased odds were high school graduate and some college in addition to living above the 200% federal poverty level. *Discussion*. Results from this study add to the limited body of the literature on determinants of substance use among Hispanic adults. Health education measures should target acculturated Hispanic adults and those with incarceration history to reduce substance use.

## 1. Background

Substance use remains a significant public health issue in the United States with an annual estimated $11 billion in national health care costs each year [1]. In 2011, 22.5 million Americans over 12 years of age (8.7% of population) reported using illicit drugs, compared to 8.3% in 2002, with the largest increase being for marijuana. Moreover, while drug use remains highest among late teenagers and those in their twenties, an increasing trend has also been noted among Americans in their fifties (6.3% in 2011 compared to 2.7% in 2002), partly attributable to the growing baby boomer generation [2].

Substance use is associated with a plethora of risky behaviors and negative health outcomes including addiction, anxiety disorders, violent behaviors, paranoia, lung disease, hypoxia, heart failure, increased risk of premature delivery, and various sexually transmitted diseases [3]. According to the Drug Abuse Warning Network (DAWN), in 2009 approximately 27% of emergency department visits were attributable to nonmedical use of pharmaceuticals and 21% due to illicit drugs, with the majority of such visits among those 21 years or older. Between 2005 and 2009, the number of drug-related emergency department visits further increased by approximately 81% (2.5 million to 4.6 million) [4]. In 2010, over 38,000 deaths in the nation were attributable to pharmaceuticals with 74% of those deaths resulting from unintentional overdose [5]. In addition, the age-adjusted death rate due to drug-induced mortality was 12.9 per 100,000 in the United States in 2010, a 2.4% increase since 2009 [5].

Though the illicit drug use rates among Hispanics are lower than the national average (6.6% versus 7.9%), a study by McCabe et al. [6] assessing the prevalence of substance use among college students noted that both Hispanic males and females had a higher percent of reported drug use as compared to other racial/ethnic groups. Similarly, the authors reported that odds of 12-month drug use were significantly higher among Hispanics as compared to African Americans and Whites. Additionally, Hispanics born in the United States have been reported to have higher rates of substance use compared to their foreign-born counterparts [7], demonstrating the potential role of acculturation on substance use outcomes.

Understanding the key determinants of substance use among the Hispanic population is critical in creating targeted interventions and lowering the socioeconomic burden associated with such behaviors. Further research is warranted among Hispanics as the population comprises the largest minority group in the nation [8]. According to the 2010 Census, the Hispanic or Latino population grew by 45% since 2000 with half of the nation's growth attributable to Hispanics [9]. As the United States' demographics continue changing, 54% of the nation is expected to be comprised of racial/ethnic minorities by 2050 [10]. Americans with Hispanic or Latino origin will comprise 30.2% of the nation's population [8].

Most studies to date, though limited to adolescent/youth populations, have highlighted several critical factors that may be associated with substance use, including religiosity, acculturation, and history of incarceration. For example, Epstein and colleagues [11] noted that linguistically acculturated Hispanic adolescents (spoke English with parents at home) were more likely to smoke any marijuana and smoke more frequently than their more traditional counterparts (spoke Spanish at home). Similarly, Vega et al. [12] noted that acculturation significantly increased drug use among Hispanic population, though a stronger effect was noted among women as compared to men. Utilizing the Hispanic Health and Nutrition Evaluation Survey, Amaro et al. [13] further noted that primarily English language use among Hispanics was significantly associated with increased marijuana and cocaine use, though mainly among those with lower education.

Johanson et al. [14] noted that among multiple drug users, youth with high religiosity (prayed, attended church functions, etc.) were less likely to initiate drugs compared to those with low religiosity. In an assessment of the association between religiosity and drug use, Chen and colleagues [15] assessed drug use prevalence among five Spanish speaking nations. Results demonstrated that increasing religiosity was associated with lower odds of first chance of tobacco use. In a study among seventh graders, Wills et al. [16] demonstrated that religiosity was negatively associated with alcohol, tobacco, and marijuana use. Moreover, Wills and colleagues noted that religiosity was also shown to serve as a buffer between life stress and initial substance use.

Recent studies have further noted that incarceration is associated with substance use. For example, a review of empirical evidence noted that drug abuse was the predominant factor leading to incarceration of women [17]. Furthermore, a recent report further noted that among the 2.3 million United States' inmates, an estimated 1.5 million have substance abuse or addiction problems [18]. However, such literature is predominantly limited to the adolescent population and does not provide a comprehensive assessment of the determinants of substance use among adult Hispanics in the United States. As such, the purpose of this study was to assess the role of religion, acculturation, and incarceration on substance use among Hispanic adults utilizing data from the National Survey on Drug Use and Health (NSDUH).

## 2. Methods

### 2.1. Study Sample

This study utilized the public use files of 2012 NSDUH, an annual survey that provides population estimates of various health outcomes and behaviors, including substance use, among noninstitutionalized Americans aged 12 and above. A multistage area probability sampling method was utilized to include residents in households, shelters, rooming houses, college dormitories, halfway houses, migrant workers, and civilians residing in military bases.

NSDUH utilizes random sampling of households across the United States followed by personal visitation to each selected household by a professional Research Triangle Institute interviewer. Upon completion of general questions, selected household residents are asked to complete an online survey and each selected individual is representative of at least 4,500 United States' residents [19]. The 2012 NSDUH response rate was reported to be approximately 73% [20]. Details of the NSDUH methodology can be obtained elsewhere [19]. Only Hispanic respondents of at least 18 years of age were included in the study, resulting in a total of 6,119 (*n*) and population estimate (*N*) of 34,808,706.

### 2.2. Measures

Recent empirical evidence has supported language of interview as a proxy for acculturation [21–24]. As such, in this study acculturation was assessed utilizing proxy measure of language of interview, previously shown to be an adequate measure of acculturation. Additionally, the influence of religion was assessed utilizing NSDUH question on whether religious beliefs influenced life. Responses were dichotomized as strongly agree/agree versus strongly disagree/disagree.

NSDUH assessed lifetime use, past year use, and past month use of any of the following substances if they were “not prescribed” and/or “took the drug only for the experience or feeling it caused”: cocaine, heroin, hallucinogens, inhalants, marijuana, pain relievers, sedatives, stimulants, and tranquilizers. Respondents reporting yes to the question were defined as substance use (lifetime, past year, and past month) in this study. Control variables included respondents' age, gender, marital status, education level, poverty, employment, and lifetime major depressive episode.

### 2.3. Data Analysis

Univariate cross-tabulations were used to compare characteristics of substance users and nonusers. Design-based F-statistics were employed to determine significant differences and weights used to obtain national population estimates. Multivariable logistic regression analyses were conducted to evaluate the effect of each independent variable on the outcome variables (lifetime, past year, and past month substance use), after adjusting for the effect of control variables, using *P* < 0.05 as the cut-off for statistical significance. A multivariable logistic regression was further conducted as a subanalysis to evaluate putative role of religion, acculturation, and incarceration on lifetime use of the most common substances. Survey-adjusted routines were used in all analyses to account for the complex sampling design. The relative contribution of each variable to substance use (lifetime, past year, and past month) among Hispanic adults was evaluated using receiver operator curve (ROC) analysis, where changes in the area under the ROC were noted after each block of variables were successively entered into the logistic regression model. Blocks were defined as basic demographics (age, gender, and marital status), socioeconomic status (poverty, education, and employment), major depressive episode, religion, acculturation, and incarceration. Nonsignificant Hosmer-Lemeshow goodness-of-fit was utilized to confirm model adequacy. SAS 9.3 was used for all statistical analyses. The study was evaluated by California State University, San Bernardino and considered exempt due to non-human subject research status.

## 3. Results

Table 1 demonstrates the distribution of sociodemographic characteristics among Hispanic adults categorized by users and nonusers of substances in lifetime, past year, and past month, with significant differences noted between the two groups (*P* < 0.05). Females were less likely to report lifetime substance use (35%) as compared to males (50%) as did those living in poverty (less than 100% FPL = 34%, 100–199% = 39%, and 200% or more = 50%). A higher percent of those who were interviewed in English (54%) reported lifetime substance use compared to those interviewed in Spanish (18%). Similarly, among those with influence of religious beliefs in life, approximately 39% reported lifetime substance use compared to significantly higher proportion (53%) among those with lower or no religious influence in life. Among those with history of incarceration, a significantly higher percent also reported lifetime substance use (78%) compared to those without any such history (36%). Similar trends were noted when assessing past year and past month substance use among Hispanic adults, though no significant differences were noted for poverty levels and employment status.

Results of logistic regression analysis, as displayed in Table 2, demonstrate a reduced likelihood of lifetime substance use among those with Spanish language interview (adjusted OR (aOR) = 0.29, 95% CI: 0.23, 0.37) and reported influence of religious beliefs in life (aOR = 0.72, 95% CI: 0.58, 0.91). On the other hand, having a history of incarceration was significantly associated with higher odds of lifetime substance use (aOR = 5.25, 95% CI: 3.94, 7.00).

Other factors associated with a lower likelihood of lifetime substance use were 65 years of age or older, female, and currently married. Additionally, factors associated with an increased likelihood of lifetime substance use were noted among those living at or above 200% FPL, increased education (high school graduate or some college), and having a lifetime major depressive episode.

Lower odds of both past year and past month substance use were associated with religiosity (past year aOR = 0.63; past month aOR = 0.59) and being less acculturated (past year aOR = 0.33; past month aOR = 0.30), while incarceration was associated with increased odds of such behavior (past year aOR = 2.45; past month aOR = 3.08). Similar to results noted in lifetime substance use, odds of past year and month substance use was positively associated with lifetime major depressive episode, while lower odds were associated with increased age, being female, and currently married.

Results of ROC/AUC analyses are presented in Table 3. Among the primary variables of interest (religion, acculturation, and incarceration), the highest relative contribution to lifetime substance use was incarceration followed by acculturation and religious influence. For substance use in past year and month, religious influence, acculturation, and incarceration had similar contributions.

Prevalence of specific substances among Hispanic adults were varied with highest lifetime use noted for marijuana (35%), followed by pain relievers (14%), cocaine (13%), and hallucinogens (11%).  Table 4 demonstrates the results of subanalysis addressing the role of religion, acculturation, and incarceration on lifetime use of such substances among Hispanic adults. Both religious influence in life and low acculturation were associated with lower odds of lifetime use of all substances analyzed (marijuana, pain relievers, cocaine, hallucinogens, and other drugs) while incarceration was associated with increased odds among Hispanic adults.

## 4. Discussion

Studies among Hispanic adults as it relates to substance use are limited, as most have elucidated such behaviors among adolescents. By utilizing a national population-based survey, we were able to demonstrate the protective effects of having religious influence in life and being less acculturated against substance use and the negative effect of incarceration among Hispanic adults.

While the majority of studies addressing religiosity and substance use have been conducted among adolescents, our study shows that an inverse relationship is also significant among Hispanic adults. Previous studies among adolescents have found religiosity to be associated with lower use of alcohol and drugs [25–29], though none were conducted among Hispanic adults. Previous studies have also noted that respondents reporting lack of sense of meaning in life were more likely to also report alcohol and drug abuse [30, 31]. In our study of Hispanic adults, a similar association between religion and substance use demonstrates the importance of addressing the construct of religiosity in future research on substance use. The underlying foundations for such an association could be the increase of nondrug related social opportunities among those with high religiosity [32], such as church attendance and religious events, though this study did not assess such a domain of religiosity.

The association between low acculturation and lower odds of substance use are consistent with previous literature demonstrating the negative effect of high acculturation on Hispanics and other minority populations in relation to various health behaviors [11, 13, 33, 34]. For example, Zemore [35] noted high acculturation to be associated with increased drinking among Hispanic women, while Gomez et al. [36] reported foreign language preference (a proxy for low acculturation) to be associated with less alcohol consumption. Such results further highlight the need for targeted prevention measures among the more acculturated Hispanics and further promoting cultural practices that help reduce the prevalence of such behaviors.

Our study also noted the positive association between history of incarceration and substance use; a potential explanation for which could be the nationwide policies on drug use, possession, and associated incarceration [37]. Such results are indicative of the need for adequate substance use treatment programs at prisons; efficacy of such programs in reducing repeat incarceration and substance use has been demonstrated [38].

Cumulatively, such results are indicative for potential scopes of targeted health promotion measures to reduce substance use burden in the nation. For example, drug use prevention measures could be targeted at more acculturated Hispanics, those with incarceration history, in addition to adults lacking nondrug associated social interactions, such as religious gatherings. Borras et al. [26] noted that while religious support group may be a barrier for those without specific religious affiliation, similar gatherings based on group support, such as drug use prevention programs equivalent to Alcoholics Anonymous, could still be valuable.

Other protective factors noted in our study include increasing age (lifetime, past year, and past month use), female gender (lifetime and past year use), and being currently married (lifetime, past year, and past month use). On the other hand, living above the 200% FPL (lifetime use only) and being a high school graduate or some college (lifetime use only) were associated with increased odds of substance use. The literature further notes similar trends in regard to other harmful behaviors. For example, binge drinking prevalence has been noted to be significantly lower among the elderly and those who are married (with no children) [39], with consistently lower rates noted among females [33, 39]. Higher odds for binge drinking were noted among those with higher socioeconomic status [33].

The literature has also reported that association between mental health status and substance use [40–42], though studies among Hispanic adults are limited. Such an association was further noted among Hispanic adults in our study demonstrating the cooccurrences of the two outcomes. Given the stigma associated with mental health among the Hispanic population [43, 44] and the results of this study highlighting the relationship to substance use, further public health efforts targeted at the population are of imperative need.

Results of this study should be interpreted with some caution due to several limitations common for survey studies. Cross-sectional data is useful for exploring associations but cannot be used to assess causal or temporal associations. Moreover, self-reported data may be susceptible to recall and social desirability biases. The small sample size of elderly Hispanics reporting past year and past month substance use limited our analysis of that age group, though it did not limit our ability to assess our question on religion, acculturation, and incarceration among all Hispanic adults. The lack of further proxies of acculturation, such as country of birth or citizenship, limited further exploration of the role of acculturation. Finally, given that homeless and institutionalized individuals were not included in NSDUH, results of this study are not generalizable to such populations.

Despite such limitations, our study contributes to the body of literature in several ways. Due to the use of a population-based survey, results of this study are generalizable to the Hispanic adult population in the United States. Though a plethora of studies have been conducted on determinants of substance use in the nation, including among Hispanic adolescents, few have assessed such a behavior and associated factors among Hispanic adults. Such studies are of necessity due to the growing Hispanic population. Our study demonstrated key protective and negative factors associated with substance use (lifetime, past year, and past month) in addition to specific commonly used substances and thus public health practitioners could further address such factors in substance use prevention measures.

Given the limited number of studies on the determinants of substance use among Hispanic adults, the current trends noted in the literature and the results of this study highlight the importance of future research and prevention for the population. In recent years, studies have also noted the importance of family cohesion in reducing substance use prevalence among adolescents [45–47]. Whether such a factor is also associated with substance use among adults, both minority and nonminority populations, and whether they can moderate the effect of religiosity, acculturation, or incarceration remains to be further evaluated.

## Figures and Tables

**Table 1:** Characteristics (sample size and weighted percent) of Hispanic adults in study sample (*n* = 6,119; *N* = 34,808,706), NSDUH 2012.

	Lifetime substance use		Substance use in the past year		Substance use in the past month	
	Yes	No	Yes	No	Yes	No
	*N* = 14,704,704	*N* = 20,104,003	*N* =5,372,864	*N* =29,435,842	*N* = 2,820,417	*N* = 31,988,289
Age (years)^abc^						
18–34	2320 (52.7)	2156 (47.3)	1226 (24.8)	3250 (75.2)	676 (13.5)	3800 (86.5)
35–49	416 (38.8)	684 (61.2)	109 (11.4)	991 (88.6)	56 (5.1)	1044 (94.9)
50–64	142 (36.5)	241 (63.5)	23 (5.9)	360 (94.1)	14 (3.8)	369 (96.2)
65 or more	20 (14.8)	140 (85.2)	5 (3.7)	155 (96.3)	1 (1.1)	159 (98.9)
Genderr^abc^						
Male	1551 (49.8)	1350 (50.2)	769 (18.2)	2132 (81.8)	443 (9.7)	2458 (90.3)
Female	1347 (34.7)	1871 (65.3)	594 (12.6)	2624 (87.4)	304 (6.5)	2914 (93.5)
Marital status^abc^						
Not currently married	2170 (47.9)	1993 (52.1)	1176 (21.8)	2987 (78.2)	663 (12.2)	3500 (87.8)
Currently married	728 (36.2)	1228 (63.8)	187 (8.6)	1769 (91.4)	84 (3.8)	1872 (96.2)
Poverty^a^						
<100% FPL	819 (34.2)	1140 (65.8)	425 (15.6)	1534 (84.4)	240 (8.7)	1719 (91.3)
100–199% FPL	859 (38.6)	1031 (61.4)	411 (14.6)	1479 (85.4)	222 (7.4)	1668 (92.6)
≥200% FPL	1190 (49.8)	1024 (50.2)	502 (15.5)	1712 (84.5)	269 (8.0)	1945 (92.0)
Education^ab^						
Less than high school	662 (27.4)	1091 (72.6)	315 (11.1)	1438 (88.9)	182 (6.4)	1571 (93.6)
High school graduate	1036 (44.9)	1090 (55.1)	509 (17.4)	1617 (82.6)	278 (9.2)	1848 (90.8)
Some college	878 (54.5)	737 (45.5)	415 (18.9)	1200 (81.1)	230 (9.0)	1385 (91.0)
College graduate	322 (49.3)	303 (50.7)	124 (14.9)	501 (85.1)	57 (8.0)	568 (92.0)
Employment status^a^						
Currently unemployed	873 (33.8)	1191 (66.2)	473 (13.9)	1591 (86.1)	274 (7.9)	1790 (92.1)
Currently employed	2025 (46.5)	2030 (53.5)	890 (16.2)	3165 (83.8)	473 (8.2)	3582 (91.8)
Lifetime major depressive episode^abc^						
Yes	505 (65.8)	230 (34.2)	264 (27.9)	471 (72.1)	131 (14.3)	604 (85.7)
No	2372 (39.5)	2959 (60.5)	1087 (14.0)	4244 (86.0)	608 (7.4)	4723 (92.6)
Religious beliefs influence life^abc^						
Strongly disagree/disagree	1129 (52.6)	857 (47.4)	640 (23.1)	1346 (76.9)	369 (13.2)	1617 (86.8)
Strongly agree/agree	1732 (38.7)	2290 (61.3)	704 (12.6)	3318 (87.4)	366 (6.2)	3656 (93.8)
Language of interview^abc^						
English	2578 (53.9)	2115 (46.1)	1266 (20.4)	3427 (79.6)	708 (10.9)	3985 (89.1)
Spanish	320 (17.5)	1106 (82.5)	97 (4.9)	1329 (95.1)	39 (2.1)	1387 (97.9)
Incarceration^abc^						
Never booked/arrested	2075 (35.5)	2956 (64.5)	942 (12.0)	4089 (88.0)	478 (5.7)	4553 (94.3)
Ever booked/arrested	816 (78.2)	252 (21.8)	419 (33.1)	649 (66.9)	268 (21.3)	800 (78.7)

FPL: federal poverty level; *n*: sample size; *N*: population estimate.

^a^Lifetime substance use *P* < 0.05;^b^past year substance use *P* < 0.05; ^c^past month substance use *P* < 0.05.

**Table 2:** Multivariable logistic regression odds ratio (and 95% CI) of substance use among Hispanic adults, NSDUH 2012.

	Lifetime substance use	Substance use in past year	Substance use in past month
Age (years) Ref. = 18–34			
35–49	0.83 (0.63, 1.10)	0.61 (0.44, 0.86)∗∗	0.55 (0.35, 0.89)∗
50–64	0.80 (0.57, 1.12)	0.26 (0.14, 0.51)∗∗∗	0.47 (0.20, 1.07)
65 or more	0.21 (0.10, 0.45)∗∗∗	NR	NR
Gender Ref. = Male			
Female	0.61 (0.51, 0.75)∗∗∗	0.74 (0.58, 0.93)∗	0.77 (0.59, 1.02)
Marital status Ref. = not currently married			
Currently married	0.79 (0.64, 0.97)∗	0.53 (0.40, 0.70)∗∗∗	0.47 (0.30, 0.73)∗∗
Poverty Ref. < 100% FPL			
100–199% FPL	1.06 (0.82, 1.37)	0.90 (0.66, 1.22)	0.83 (0.53, 1.28)
≥200% FPL	1.48 (1.15, 1.90)∗∗	0.95 (0.72, 1.25)	0.97 (0.62, 1.54)
Education Ref. = less than high school			
High school graduate	1.32 (1.06, 1.64)∗	1.05 (0.73, 1.51)	0.96 (0.65, 1.42)
Some college	1.54 (1.21, 1.95)∗∗	0.97 (0.71, 1.33)	0.80 (0.53, 1.20)
College graduate	1.32 (0.88, 1.98)	0.93 (0.60, 1.45)	0.90 (0.43, 1.86)
Employment status Ref. = currently unemployed			
Currently employed	1.22 (1.00, 1.47)∗	0.96 (0.74, 1.26)	0.84 (0.60, 1.18)
Lifetime major depressive episode Ref. = No			
Yes	2.97 (2.03, 4.33)∗∗∗	2.22 (1.58, 3.13)∗∗∗	1.75 (1.09, 2.83)∗
Religious beliefs influence life Ref. = strongly disagree/disagree			
Strongly agree/agree	0.72 (0.58, 0.91)∗	0.63 (0.49, 0.82)∗∗	0.59 (0.43, 0.81)∗∗
Language of interview Ref. = English			
Spanish	0.29 (0.23, 0.37)∗∗∗	0.33 (0.22, 0.51)∗∗∗	0.30 (0.17, 0.53)∗∗∗
Incarceration Ref. = never booked/arrested			
Ever booked/arrested	5.25 (3.94, 7.00)∗∗∗	2.45 (1.85, 3.24)∗∗∗	3.08 (2.21, 4.29)∗∗∗

Ref.: reference category; FPL: federal poverty level; CI: confidence interval; NR: not reported due to low sample size.

*P* <0.05∗, <0.005∗∗, and <0.0001∗∗∗.

**Table 3:** Receiver operator curve (ROC) analysis of substance use logistic regression models^a^.

	Lifetime substance use		Substance use in past year		Substance use in past month	
	Area under curve	Gain in AUC	Area under curve	Gain in AUC	Area under curve	Gain in AUC
No variables (random)	0.5		0.5		0.5	
Adding demographics	0.615	0.115	0.684	0.184	0.681	0.181
Adding socioeconomic status	0.641	0.026	0.692	0.008	0.689	0.008
Adding major depressive episode	0.667	0.026	0.710	0.018	0.696	0.007
Adding religious influence in life	0.675	0.008	0.727	0.017	0.715	0.019
Adding acculturation	0.706	0.031	0.744	0.017	0.739	0.024
Adding incarceration	0.745	0.039	0.762	0.018	0.762	0.023

^a^Results from successively adding in each block of variables. The final result represents the full models shown in Table 2.

**Table 4:** Multivariable logistic regression analysis of lifetime use of specific substances among Hispanic adults, NSDUH 2012.

	Marijuana	Pain relievers	Cocaine	Hallucinogens	Other drugs
Religious beliefs influence life					
Strongly disagree/disagree (ref.)					
Strongly agree/agree	0.74 (0.58, 0.95)∗	0.94 (0.68, 1.28)	0.69 (0.50, 0.96)∗	0.67 (0.51, 0.87)∗∗	0.86 (0.65, 1.14)
Acculturation					
English (ref).					
Spanish	0.17 (0.12, 0.24)∗∗∗	0.48 (0.32, 0.72)∗∗	0.43 (0.26, 0.69)∗∗	0.10 (0.06, 0.20)∗∗∗	0.36 (0.24, 0.55)∗∗∗
Incarceration					
Never booked/arrested (ref.)					
Ever booked/arrested	5.78 (4.26, 7.84)∗∗∗	2.37 (1.77, 3.17)∗∗∗	4.49 (3.24, 6.22)∗∗∗	4.64 (3.45, 6.23)∗∗∗	3.73 (2.67, 5.20)∗∗∗

Other drugs include heroin, inhalants, sedatives, stimulants, and tranquilizers. Ref.: reference category. Model adjusted for age, gender, marital status, poverty, education, employment, and lifetime major depressive episodes.

*P* < 0.05∗, <0.005∗∗, and <0.0001∗∗∗.
